# Association between immigration enforcement encounters and COVID-19 testing and delays in care: a cross-sectional study of undocumented young adult immigrants in california

**DOI:** 10.1186/s12889-022-13994-0

**Published:** 2022-08-16

**Authors:** May Sudhinaraset, Hye Young Choi, Ezinne Nwankwo, Maria-Elena De Trinidad Young

**Affiliations:** 1 Fielding School of Public Health, Community Health Sciences Jonathan and Karin, University of California Los Angeles, Los Angeles, CA USA; 2 Department of Social and Behavioral Sciences, Harvard T.H. Chan School of Public Health, Boston, MA USA; 3 Department of Public Health, School of Social Sciences, Humanities, and Arts, University of California Merced, Merced, CA USA

**Keywords:** COVID-19 testing, Immigration enforcement, Health services accessibility, Healthcare disparities, Emigration and immigration, Undocumented immigrants, Asian Americans, Hispanic American

## Abstract

**Background:**

Undocumented immigrants are expected to face increased risks related to COVID-19 due to marginalizing restrictive immigration policies. However, few studies have assessed the prevalence of direct encounters with the immigration enforcement system among the undocumented and its impacts on their COVID-related health behaviors and outcomes. In this study, we quantify undocumented immigrants' lifetime exposure to various immigration enforcement tactics and their association with delays in COVID-19 testing and healthcare behaviors.

**Methods:**

This cross-sectional study included a non-random sample of 326 Asian and Latinx undocumented immigrants in California from September 2020 to February 2021. The primary exposure was immigration enforcement encounter scores ranging from 0–9, assessed through self-reports of direct experiences with the immigration system, immigration officials, and law enforcement. The main outcomes were positive test for COVID-19, had or suspected having COVID-19, and delayed or avoided testing and/or treatment for COVID-19 due to immigration status. We used multivariable logistic regression models to examine the association between the primary exposure and outcomes of interest.

**Results:**

Among 326 participants, 7% had received a positive COVID-19 test result, while 43% reported having or suspected having COVID-19. Almost 13% delayed or avoided COVID-19 testing and/or treatment because of their immigration status. Overall, an increase in immigration enforcement encounters was associated with higher odds of suspecting having had COVID-19 (aOR = 1.13; 95% CI: 1.01,1.26). Reporting an additional enforcement encounter was associated with higher odds of delaying or avoiding testing and/or treatment because of immigration status (aOR = 1.53, 95% CI: 1.26,1.86). Compared to their Latino counterparts, Asian respondents were more likely to report higher odds of delaying or avoiding testing and/or treatment (aOR = 3.13, 95% CI: 1.17,8.42). There were no significant associations between the enforcement score and testing positive for COVID-19. Additionally, while Latinxs were more likely to report immigration enforcement encounters than Asians, there were no differences in the effects of race on COVID-19 testing and healthcare behaviors in models with race as an interaction term (*p* < 0.05).

**Conclusions:**

Immigration enforcement encounters compound barriers to COVID-19 testing and treatment for undocumented immigrants.

## Introduction

The coronavirus disease 2019 (COVID-19), caused by the SARS-CoV-2 novel coronavirus, has exacerbated long-standing social and health inequities related to structural racism, particularly among immigrant communities [1, 2]. There are over 44.9 million immigrants in the US [3], 10.7 million who are undocumented, and 8 million citizens who live in a household with an undocumented family member [4]. Immigrants experience disproportionate COVID-related morbidities and mortality compared to those who are US-born [5, 6]. National COVID reporting statistics do not disaggregate by country of origin, but undocumented immigrants are expected to report disproportionately high levels of COVID-related morbidities and mortalities given their higher representation as essential workers, historical mistreatment resulting in medical mistrust, and fear from immigration enforcement [2, 7]. However, there is a critical lack of COVID-related data on this population.

Undocumented immigrants are a critical group, not only because of the size of this population, but because of the growing evidence that immigration enforcement policies—which target undocumented immigrants—are detrimental to health [8, 9]. Immigration enforcement policies perpetuate “violence of uncertainty” by creating systematic insecurity and threatening the daily lives of immigrants [10]. However, most studies have focused on acute events, such as an immigration raid [11] or changes in political elections [12], single policies [13], or overall immigrant climate [14]. Few studies have examined immigrants’ direct encounters with the immigration enforcement system, which targets immigrants through a range of harmful tactics spanning surveillance, policing, and deportation [15, 16]. Studies are also limited on how immigration enforcement influences undocumented immigrants’ COVID-related health behaviors [17–19] and acceptability of COVID-19 vaccines [20]. These studies found that immigrant-related fears and anti-immigrant rhetoric resulted in delays in COVID-19 treatment and undermined contact tracing efforts [17–19]. However, these studies focus on Latinx populations or use qualitative data; it is unclear whether these results also extend to the 1.5 million undocumented Asian immigrants, and the extent to which undocumented Asians encounter immigration enforcement activities. Given that Asians are the fastest growing undocumented group and experience unique racialized stressors during the COVID-19 pandemic, including an increase in anti-Asian racism and xenophobic attacks [21, 22], it is important to assess how immigration enforcement influences their behaviors and health outcomes. This study examines the association between immigration enforcement encounters and COVID-19 testing and treatment.

## Methods

### Study sample and recruitment

This paper uses survey data collected between September 2020 to February 2021 from the COVID-19 BRAVE (Building community Raising All immigrant Voices for health Equity) Study, which aimed to examine the social, economic, and health impacts of COVID-19 among undocumented young adult immigrants in California. This study followed the Strengthening the Reporting of Observational Studies in Epidemiology (STROBE) reporting guidelines. Our study was approved by the Institutional Review Board at the University of California, Los Angeles.

We used a community-engaged approach by consulting a Community Advisory Board (CAB) composed of undocumented individuals and experts in health policy, education, and immigrant advocacy throughout survey development, recruitment, and data interpretation processes. Participants were recruited through community and school-based organizations with established histories of serving undocumented immigrants. Community partners recruited through their email list serves and flyers; additionally, to minimize potential selection bias for immigrants embedded in partner networks, instructions on accessing password-protected surveys were also advertised on social media. Each community partner provided a unique password for the survey to minimize fraudulent participation. Participants received a $10 gift card for participation. Emails were delinked from study data. Screening and surveys were administered online through Qualtrics. Eligible participants were: 1) undocumented; 2) Asian and/or Latinx; 3) aged 18–39 years; 4) live in California; and 5) able to take a 15-min online survey in English or Spanish. An original goal of the overall BRAVE Study was to examine how Deferred Action for Childhood Arrivals (DACA) influences immigrant health. DACA is a major immigration policy, an Executive Order signed by the Obama Administration in 2012, which granted certain undocumented young adults up to age 30 years the right to work and live in the US. As 2022 marks the 10^th^ anniversary of DACA, we include up to age 39 years to include the earliest DACA recipients. All participants provided informed consent and all participants chose to take the survey in English.

A non-random sample of 438 participants were emailed a link to the survey, and 366 respondents completed the survey (83.5% participation rate). An additional 163 were screened but ineligible. Post-validation checks were performed including assessments of duration of time taken to complete survey (those who spent < 5 min on survey were excluded), assessment of concordance of items that follow logical flow (i.e. established DACA eligibility requirements met for those who have DACA (i.e., currently a DACA recipient [or in renewal pending status], expired lawful immigration as of June 15, 2012, living in the U.S. for at least 5 years prior to June 15, 2012, and not having a felony or a significant misdemeanor conviction). We excluded 24 participants who did not pass these validation checks. We have applied similar procedures to ensure sample validity in other studies [23, 24]. We excluded an additional 16 respondents who did not complete one or more questions included in these analyses. Missing variables are minimal and all less than 3%. The final analytic sample included 326 participants.

### Outcome measures

Respondents were asked the following questions about possible illness and testing: (a) Have you ever had, or thought you might have had, the Coronavirus, COVID-19? (referred to as had/suspected having COVID-19) (b) Did you ever receive a positive test result for COVID-19? (tested positive) (c) Did you delay or avoid getting tested and/or seeking treatment for COVID-related symptoms because of your immigration status? (avoided testing). Each question was a binary yes/no response.

### Exposure measures

Immigration enforcement encounters were the primary exposures of interest. Respondents were asked to indicate their encounters with the immigration system, immigration authorities, and law enforcement by reporting if: (a) there was ever a time they decided not to apply for one or more non-cash government benefits because of worries it would disqualify them, or a family member, from obtaining a green card or becoming a U.S. citizen; (b) they or someone they know experienced an immigration raid at work or at home; (c) someone they know had ever been detained or deported by immigration authorities; (d) they had ever faced deportation proceedings; (e) there was ever a time they decided not to leave their house or stayed away from certain areas to avoid the police or immigration authorities; (f) there was ever a time they decided to avoid travelling by car, bus, train, or plane to avoid internal checkpoints or TSA authorities; (g) they had ever been watched by a law enforcement officer on the street or a public place; (h) they had ever been stopped for no good reason by law enforcement; (i) they had ever been asked to show proof of their citizenship or legal status by a police officer or other law enforcement authority; (k) they had seen immigration authorities in their neighborhood, and if (k) they fear getting deported, reported as “all of the time,” “most of the time,” “some of the time,” and “no, I do not.” This question was dichotomized as 0 (no, I do not) and 1 (all/most/some of the time). All other immigration enforcement encounter questions were a binary yes/no response. The first question regarding applying for public benefits measures fears of the public charge rule. Under the Public Charge rule, those accessing public benefits and deemed likely or liable to become a public charge may be denied visas or inadmissible to the US. [9] immigration enforcement score was created using respondents’ total affirmative responses to each question (mean = 3.52, SD = 2.06) and ranged from 0–9, with higher scores indicating more immigration enforcement encounters. These questions were adapted from the Research on Immigrant Health and State Policy Study survey which aimed to develop measures of immigration enforcement experiences, including surveillance, policing, and deportatioon [25]. These measures have been used in other studies with undocumented immigrants [20, 26].

### Covariates

Independent variables were comprised of demographic, socioeconomic, and health insurance measures. Demographic characteristics, included gender, which was reported as female and male. Race and ethnicity were reported as Latino or Asian, DACA status was coded as no DACA or DACA, and age was a categorical variable, 18–24, 25–30 and 31 + years. Socioeconomic variables included highest level of education, reported as high school or less than high school, some college/community college, and college or graduate school. Respondents indicated yes or no to questions about their employment status, school enrollment, and speaking English at home. Respondents reported their health insurance status and were asked to specify if it was a county health plan, Medi-Cal, school health plan, private/employee health plan or other health insurance. This categorical variable was dichotomized, so that “no” represented uninsured and “yes” included all other responses.

### Statistical analysis

Descriptive and bivariate analyses were conducted to examine means, frequencies, and associations. We used multivariable logistic regression models to assess the association between immigration enforcement encounters and COVID-19 testing and treatment outcomes, adjusting for covariates. We included covariates that were theoretically associated with our main predictor and outcomes of interest. We then assessed bivariate associations using a p-value cut-off of 0.2. For a few covariates, such as gender, education level, and currently in school, we included these variables based on theoretical and conceptual importance even if they did not meet the 0.2 p-value cut-off. Finally, we assessed for collinearity using a variance inflation factor. We fit interaction models to assess differences between immigration enforcement encounters and the outcomes by race; however, we do not report those findings as there were no statistically significant results. We conducted sensitivity analyses where we used the continuous immigration enforcement score as a binary measure: (a) cut off at the mean (b) cut off at the median; and as (c) zero or one or more enforcement encounters. We observed similar overall patterns, net of covariates. Reporting one or more immigration enforcement encounters was significantly associated with higher odds of suspecting COVID-19 and reporting three or more encounters was significantly associated with higher odds of delaying or avoiding testing and/or seeking treatment for COVID-related symptoms because of immigration status [data not shown, available upon request]. All analyses were conducted using Stata version 15 and statistical significance was set at *p* < 0.05.

## Results

Among 326 study participants, the majority were female (75%) and most were between ages 18–24 years (74%). Close to half of the sample reported being employed (49%) and over a third had completed a college degree or higher (34%). Most participants were enrolled in school (88%), spoke English at home (85%) and had health insurance (78%). Overall, 7% had received a positive COVID-19 test result, and almost half of the study sample reported having or suspecting COVID-19 (43%). Over 10% avoided testing and/or seeking COVID-19 treatment because of their immigration status (13%) (Table 1).

**Table 1:** Characteristics of the study sample, *N* = 326

Sample characteristics	No. (%)
Gender	
Female	245 (75)
Male	81 (25)
Race/ethnicity	
Latino	278 (85)
Asian	48 (15)
DACA status	
No DACA	117 (36)
DACA	209 (64)
Age (M (SD))	22.62 (3.99)
18–24	241 (74)
25–30	71 (22)
31 +	14 (4)
Highest level of education	
High school or less than high school	77 (24)
Some college/community college	138 (42)
College or graduate school	111 (34)
Employed	
No	166 (51)
Yes	160 (49)
Enrolled in school	
No	40 (12)
Yes	286 (88)
Speaks English at home	
No	48 (15)
Yes	278 (85)
Health insurance	
Uninsured	71 (22)
County health plan	13 (4)
Medi-Cal	131 (40)
School health plan	63 (19)
Private/employee health plan	42 (13)
Other	6 (2)
Health insurance	
No	71 (22)
Yes	255 (78)
Had (or suspected) COVID-19	
No	187 (57)
Yes	139 (43)
Received a positive test result for COVID-19	
No	302 (93)
Yes	24 (7)
Delayed/avoided getting tested and/or seeking COVID-19 treatment because immigration status	
No	285 (87)
Yes	41 (13)

Note. *M*, Mean, *SD*, Standard Deviation

Most respondents reported at least one immigration enforcement encounter (91%), with respondents reporting an average of 3.52 (SD = 2.06) (Table 2). Latinos had a higher mean number of immigration enforcement encounters. Specifically, Latinos were significantly more likely to report avoiding traveling to evade checkpoints or TSA authorities compared to Asians (71% vs. 56%) and more likely to report ever being watched by law enforcement on the street or a public place compared to Asians (24% vs. 8%). Similarly, Latinos were more likely to report seeing immigration authorities in their neighborhood compared to Asians (23% vs. 8%). Latinos were significantly more likely to report knowing someone who had been detained or deported compared to Asian respondents (50% vs. 27%). About 39% of Latinos reported fear of deportation compared to 25% of Asian respondents. There were no differences among Latino and Asian respondents in regards to other immigration enforcement encounter indicators. For example, over half of the sample indicated that there was a time they did not apply for non-cash government benefits because of worries they or their family member would be deemed a public charge (53%). About one in six had experienced an immigration raid at work or at home (16%). Few respondents reported previously facing deportation proceedings (1%). Almost 9% indicated that they had been stopped for no good reason by law enforcement and even fewer had been asked by police officers or other law enforcement authority to show proof of their citizenship or legal status (4%).

**Table 2:** Immigration enforcement encounters, *N* = 326

Variables	Asians (No. (%))	Latinx (No. (%))	Total (No. (%)	*P* Value
	48 (15)	278 (85)	326 (100)	
Immigration enforcement encounters count (0–9) (M (SD))	2.73 (2.12)	3.66 (2.02)	3.52 (2.10)	0.004
Immigration enforcement encounters				0.007
None	9 (19)	19 (7)	28 (9)	
More than 1 (1 +)	39 (81)	259 (93)	298 (91)	
Did not apply for non-cash benefits				0.470
No	25 (52)	129 (46)	154 (47)	
Yes	23 (48)	149 (54)	172 (53)	
Experienced an immigration raid				0.830
No	40 (83)	235 (85)	275 (84)	
Yes	8 (17)	43 (16)	51 (16)	
Someone you know ever been detained or deported				0.003
No	35 (73)	139 (50)	174 (53)	
Yes	13 (27)	139 (50)	152 (47)	
Faced deportation proceedings				0.360
No	47 (98)	276 (99)	323 (99)	
Yes	1 (2)	2 (1)	3 (1)	
Fears getting deported				0.060
No	36 (75)	169 (61)	205 (63)	
Yes	12 (25)	109 (39)	121 (37)	
Did not leave home				0.240
No	15 (31)	65 (23)	80 (25)	
Yes	33 (69)	213 (77)	246 (76)	
Avoided travelling by car, bus, train				0.038
No	21 (44)	80 (29)	101 (31)	
Yes	27 (56)	198 (71)	225 (69)	
Been watched by law enforcement on the street				0.015
No	44 (92)	211 (76)	255 (78)	
Yes	4 (8)	67 (24)	71 (22)	
Been stopped for no good reason by law enforcement				0.950
No	44 (92)	254 (91)	298 (91)	
Yes	4 (8)	24 (9)	28 (9)	
Been asked to show proof of citizenship or legal status				0.850
No	46 (96)	268 (96)	314 (96)	
Yes	2 (4)	10 (4)	12 (4)	
Has seen immigration authorities in neighborhood				0.021
No	44 (92)	214 (77)	258 (79)	
Yes	4 (8)	64 (23)	68 (21)	

In adjusted models, an increase in immigration enforcement encounters was significantly associated with a 1.13 times higher odds of reporting having had or suspecting COVID-19 (95% CI: 1.01,1.26; *p* = 0.036) (Table 3). An increase in immigration enforcement encounters was significantly associated with higher odds of delaying or avoiding getting tested and/or seeking treatment for COVID-related symptoms because of immigration status (aOR = 1.53, 95% CI: 1.26,1.86). There were no significant associations between immigration enforcement encounters and testing positive for COVID-19. Compared to their Latino counterparts, Asian respondents were significantly more likely to report higher odds of delaying or avoiding getting tested (aOR = 3.13, 95% CI: 1.17,8.42). Speaking English at home was marginally associated with higher odds of reporting such delays or avoidance (aOR = 3.00, 95% CI: 0.83,10.88; *p* = 0.094). There were no statistically significant results for the interaction between race and immigration enforcement on COVID-related testing and treatment.

**Table 3:** Associations between immigration enforcement score and COVID-19 testing and treatment

Variables	COVID-19 Suspect		COVID-19 Positive		COVID-19 Delayed	
	aOR	95% CI	aOR	95% CI	aOR	95% CI
Immigration enforcement score (0–9)	1.13*	1.01,1.26	0.94	0.76,1.16	1.53***	1.26,1.86
Gender						
Female	0.88	0.51,1.50	1.09	0.40,3.00	0.83	0.36,1.91
Male	1.00	1.00,1.00	1.00	1.00,1.00	1.00	1.00,1.00
Race/ethnicity						
Latino	1.00	1.00,1.00	1.00	1.00,1.00	1.00	1.00,1.00
API	1.14	0.57,2.26	0.46	0.10,2.23	3.13*	1.17,8.42
DACA status						
No DACA	1.00	1.00,1.00	1.00	1.00,1.00	1.00	1.00,1.00
DACA	1.41	0.81,2.44	1.18	0.41,3.42	0.84	0.37,1.90
Age						
18–24	1.00	1.00,1.00	1.00	1.00,1.00	1.00	1.00,1.00
25–30	0.59	0.31,1.12	0.68	0.19,2.44	0.51	0.17,1.52
31 +	0.75	0.23,2.43	2.86	0.63,12.91	1.00	1.00,1.00
Highest level of education						
High school or less than high school	1.00	1.00,1.00	1.00	1.00,1.00	1.00	1.00,1.00
Some college/community college	0.96	0.52,1.78	1.04	0.31,3.48	1.19	0.50,2.87
College or graduate school	0.94	0.44,2.02	1.13	0.27,4.69	0.46	0.12,1.77
Employed						
No	1.00	1.00,1.00	1.00	1.00,1.00	1.00	1.00,1.00
Yes	1.50	0.90,2.49	1.24	0.47,3.28	0.85	0.39,1.86
Enrolled in school						
No	1.00	1.00,1.00	1.00	1.00,1.00	1.00	1.00,1.00
Yes	0.86	0.37,1.96	0.55	0.13,2.32	0.35	0.08,1.52
Speaks English at home						
No	1.00	1.00,1.00	1.00	1.00,1.00	1.00	1.00,1.00
Yes	2.27*	1.13,4.58	1.18	0.33,4.29	3.00 +	0.83,10.88
Health insurance						
No	1.00	1.00,1.00	1.00	1.00,1.00	1.00	1.00,1.00
Yes	1.11	0.62,1.97	3.01	0.67,13.61	0.70	0.31,1.58
Constant	0.21*	0.05,0.85	0.04*	0.00,0.65	0.05*	0.00,0.54
-2 Log Likelihood	-213.72		-81.21		-105.08	
AIC	453.43		188.42		234.17	
BIC	502.66		237.65		279.09	
N. of cases	326		326		312	

Note. *aOR*, Adjusted Odds Ratio

+* p* < 0.10, * *p *< 0.05, ** *p *< 0.01, *** p < 0.001

When examining specific experiences of immigration enforcement encounters, experiencing a raid was significantly associated with higher odds of having had or suspecting COVID-19 (aOR = 2.32, 95% CI: 1.13, 4.74) (Table 4). Seeing immigration authorities in the neighborhood was marginally associated with higher odds of having had or suspecting COVID-19 (aOR = 1.83, 95% CI: 0.98,3.41), but was significantly associated with delaying or avoiding getting tested and/or seeking treatment for COVID-related symptoms because of immigration status (aOR = 2.63, 95% CI: 1.14,6.06). Those who reported a time when they decided not to apply for one or more non-cash government benefits because of public charge fears had significantly higher odds of delaying or avoiding getting tested and/or seeking treatment for COVID-related symptoms because of their immigration status (aOR = 2.69, 95% CI: 1.18,6.12). There were no significant associations between the individual immigration enforcement measures and testing positive for COVID-19.

**Table 4:** Associations between immigration enforcement encounters and COVID-19 testing and treatment

Variables	COVID-19 Suspect		COVID-19 Positive		COVID-19 Delayed	
	aOR	95% CI	aOR	95% CI	aOR	95% CI
Immigration enforcement encounters						
Ever a time when did not apply for non-cash benefits	0.79	0.48,1.29	0.95	0.38,2.37	2.69*	1.18,6.12
Experienced an immigration raid	2.32*	1.13,4.74	2.14	0.60,7.66	1.85	0.74,4.66
Someone you know ever been detained or deported	0.85	0.50,1.44	0.89	0.33,2.38	0.99	0.42,2.32
Faced deportation proceedings	1.80	0.12,27.01	1.00	1.00,1.00	1.00	1.00,1.00
Fears getting deported	1.00	0.59,1.71	0.66	0.23,1.90	1.28	0.56,2.92
Did not leave home	1.55	0.80,3.01	0.89	0.27,2.92	2.61	0.66,10.34
Avoided travelling by car, bus, train	1.27	0.71,2.28	0.75	0.25,2.21	1.59	0.58,4.36
Been watched by law enforcement on the street	0.62	0.33,1.19	1.49	0.49,4.51	1.92	0.77,4.76
Been stopped for no good reason by law enforcement	0.84	0.34,2.08	1.46	0.33,6.46	0.31	0.06,1.65
Been asked to show proof of citizenship or legal status	1.42	0.40,5.06	1.00	1.00,1.00	0.79	0.12,5.20
Has seen immigration authorities in neighborhood	1.83 +	0.98,3.41	0.48	0.12,1.89	2.63*	1.14,6.06
Constant	0.16*	0.04,0.69	0.05*	0.00,0.80	0.03**	0.00,0.39
-2 Log Likelihood	-207.04		-78.18		-98.09	
AIC	460.08		198.37		238.18	
BIC	547.18		276.9		316.58	
N. of cases	326		311		309	

aOR, Adjusted Odds Ratio

+ *p* < 0.10, * *p* < 0.05, ** *p* < 0.01, *** *p* < 0.001

## Discussion

This study found a relationship between Latino and Asian immigrants’ immigration enforcement encounters and possible COVID-19 illness and avoidance of COVID-19 testing and treatment. Specifically, a greater number of encounters with the immigration system, immigration authorities, or law enforcement was associated with reporting having had or suspecting having had COVID-19 and delaying testing and treatment because of immigration status. Specific encounters, such as a raid and seeing immigration authorities in neighborhood were significantly associated with higher odds of having had or suspecting to have had COVID-19; while fear related to public charge and seeing immigration authorities in neighborhood was significantly associated with higher odds of delaying or avoiding COVID testing or treatment.

Overall, a substantial proportion of our sample contended with the daily stressors of the immigration enforcement system, with over 90% of the sample reporting at least one enforcement encounter. These are some of the first comparative data showing how common enforcement encounters are among two of the nation’s largest immigrant groups. While there were differences in the patterns of these encounters between Latino and Asian respondents, both groups reported non-negligible levels of contact with these restrictive aspects of immigration policy. In general, a higher proportion of Latinos reported each of the encounters; but there were also notable similarities. Both groups reported similar levels of being concerned about public charge and having been racially profiled. Our observed patterns of enforcement exposures likely result from historical racialization processes of “illegality” and “legality” among and within different immigrant groups, which involve ostensibly race-neutral immigration laws, enforcement practices, as well as reifying narratives of undocumented immigrants in media and society [27].

Our findings broaden understanding of the intersecting structural risks that Latino and Asian immigrants experienced in the context of both the pandemic and restrictive immigration policy. First, greater exposure to immigration enforcement policies may correspond with the various structural, institutional, and individual risks for coronavirus infection [28]. This cross-sectional analysis cannot establish a temporal or causal relationship, but highlights the compounding impacts of both enforcement policy and COVID-19 related inequities. The most concrete intersection of these risks has been observed in immigration detention centers, where immigrants have been at extremely high risk of infection [29, 30]. This patterning of risk, similarly, likely explains the findings in our community-based sample. For example, although ICE ostensibly changed enforcement priorities, there is evidence that immigration enforcement continued unabated during the pandemic [31] and many of the most exposed essential workers, such as those in agriculture, are also immigrants and disproportionately undocumented [32]. Additionally, in the first year of the pandemic, COVID-19 stimulus funds were limited to families in which all adults had a social security number. Therefore, individuals without a social security number or those married to individuals without a social security number were unable to access these economic benefits. This contributes to the economic vulnerability of the undocumented community and further deepens health inequities for this group.

Second, our findings suggest that exposure to enforcement may be associated with concerns about accessing critical health services, even COVID-19 related resources (e.g., testing, vaccines) that are free of charge and not limited by legal status. This is consistent with evidence that immigrants who reside in communities with more enforcement policies or higher levels of enforcement actions are more likely to withdraw from public benefits, lack a usual source of care, and delay seeking care [33–35]. In addition, the findings may be explained by the implementation of the draconian public charge rule during the pandemic. Even prior to its full implementation, the public charge rule was associated with a reduction in public insurance rates among Latino families [36]. The delay in COVID-19 testing and treatment may be one of the many consequences of the “chilling effect” that this rule has had among immigrants. Immigration enforcement policies perpetuate “violence of uncertainty” by redefining how immigrants are able to live their daily lives, resulting in health inequities due to fear and medical mistrust [10, 23, 37]. While this specific study focuses on COVID-19 testing and treatment behaviors, other health outcomes such as vaccine acceptability and uptake and other health behaviors may follow similar patterns [20]. This points to lasting effects of immigration enforcement on immigrant health behaviors and public health overall.

Finally, while immigration enforcement was associated with reporting having had or suspecting having had COVID-19, there were no similar associations with testing positive for COVID-19. This is likely a reflection of the barriers to testing. Indeed, asking respondents if they had an affirmative test-positive is likely an undercount of the impact of COVID-19 illness. Future research is needed to assess how enforcement exposure may be linked to other intervening risk factors, such as worry about employer immigration-related retaliation at work or concern about seeking health services.

The study had some limitations. First, as a non-random cross-sectional study, the findings cannot establish temporal or causal relationships between enforcement and COVID-related risks and limits generalizability. Second, confirmed and suspected COVID-19 reports were not defined in a set period of time; therefore, COVID-19 cases increased over time. Third, the sample of Asian respondents was small (*n* = 48, 15% of sample); however, at 15% it is proportionate to the undocumented Asian immigrant population in California.

While our sample size does not support additional granularity in race/ethnicity groupings, future studies should recruit larger samples of undocumented immigrants to examine different ethnicities (e.g. Southeast Asian, South Asian, East Asian) and sub-ethnicities (e.g. Afro-Latino, Afro-Caribbean, Latinasian, Indigenous). Given within-group disparities among Asians and Latinx populations that likely underlie enforcement and COVID-19 risks, we also recommend researchers report disaggregated data whenever possible.

Additionally, future studies should offer in-language surveys for multiple Asian groups. The lack of Asian language surveys in our study may have contributed to the limited participation by Asians. It should be noted, however, that while the survey was offered in Spanish and English, all participants chose to take the survey in English, which may be reflective of the more educated and connected status of our participants.

## Conclusions

In the last several years, immigrant communities across the nation have simultaneously contended with the expansion of restrictive immigration policies and the onset of the pandemic – both of which have resulted in disproportionate negative impacts in communities of color. This study provides insights into immigrants’ intersecting structural health risks. Policy changes are needed to end enforcement practices that create barriers and to ensure accessibility to COVID-19 testing and treatment that is responsive to immigrants who have already contended with the harms of enforcement.

## Data Availability

Data that support the findings of this study are unavailable due to the sensitivity of the study population. Data is available upon request and approval from the lead author.
